# *OsmiR535*, a Potential Genetic Editing Target for Drought and Salinity Stress Tolerance in *Oryza sativa*

**DOI:** 10.3390/plants9101337

**Published:** 2020-10-10

**Authors:** Erkui Yue, Huan Cao, Bohan Liu

**Affiliations:** 1 Institute of Crop Science, College of Agriculture and Biotechnology, Zhejiang University, Hangzhou 310058, China; erkuiyue@zju.edu.cn (E.Y.); 21816122@zju.edu.cn (H.C.); 2 Southern Regional Collaborative Innovation Center for Grain and Oil Crops in China, College of Agriculture, Hunan Agricultural University, Changsha 410128, China

**Keywords:** miRNA, salinity tolerance, drought tolerance, OsmiR535, CRISPR/Cas9, *Oryza sativa* L.

## Abstract

*OsmiR535* belongs to the miR156/miR529/miR535 superfamily, a highly conserved miRNA family in plants. *OsmiR535* is involved in regulating the cold-stress response, modulating plant development, and determining panicle architecture and grain length. However, the role that *OsmiR535* plays in plant responses to drought and salinity are elusive. In the current study, molecular and genetic engineering techniques were used to elucidate the possible role of *OsmiR535* in response to NaCl, PEG(Poly ethylene glycol), ABA(Abscisic acid), and dehydration stresses. Our results showed that *OsmiR535* is induced under stressed conditions as compared to control. With transgenic and CRISPR/Cas9 knockout system techniques, our results verified that either inhibition or knockout of *OsmiR535* in rice could enhance the tolerance of plants to NaCl, ABA, dehydration and PEG stresses. In addition, the overexpression of *OsmiR535* significantly reduced the survival rate of rice seedlings during PEG and dehydration post-stress recovery. Our results demonstrated that *OsmiR535* negatively regulates the stress response in rice. Moreover, our practical application of CRISPR/Cas9 mediated genome editing created a homozygous 5 bp deletion in the coding sequence of *OsmiR535*, demonstrating that *OsmiR535* could be a useful genetic editing target for drought and salinity tolerance and a new marker for molecular breeding of *Oryza sativa*.

## 1. Introduction

MicroRNAs (miRNAs) are a class of 18–24 nt non-coding RNAs that combine with AGO(ARGONAUTE) proteins to form the miRNA-induced silencing complex (miRISC), which can recognize and inhibit the expression of target genes at the post-transcription level [1,2]. There is growing evidence that miRNAs can influence various biological processes, such as plant growth and development, the stress response, and metabolic processes [3,4,5]. *miR156*, *miR529*, and *miR535* share high sequence similarity, belong to the miR156/miR529/miR535 superfamily, and play an essential role in modulating plant growth and development and SPLs(SQUAMOSA promoter-binding protein-like) expression [6,7,8]. Unlike *OsmiR156*s and *OsmiR529*s, of which are twelve and two copies in rice, respectively, there is only one *OsmiR535* in the rice genome [7].

The *OsmiR535* role in abiotic stresses was firstly reported in a microarray study, which demonstrated that *OsmiR535* could be induced by cold-stresses [9]. Recent studies revealed the *OsmiR535* negatively regulates cold tolerance in rice, as the overexpression of *OsmiR535* aggravated cold-induced cell death and ROS(Reactive oxygen species) accumulation [10]. *OsmiR535* modulates plant height, panicle architecture, and grain length by suppressing the expression of *OsSPL7/12/16* [6]. However, the involvement of *OsmiR535* in response to drought (dehydration and PEG-induced), ABA, and salinity stress in rice is elusive.

The objective of our study was to investigate *OsmiR535* function in drought and salinity responses in rice by using different genetic engineering techniques. In the present study, we validated the expression pattern of *OsmiR535* and its putative target genes by real-time qPCR. We also used CRISPR/Cas9, STTM(Short tandem target mimic), and UBI(Ubiquitin) promoter-driven overexpression techniques to genetically manipulate *OsmiR535* and generate transgenic lines. Then, we tested transgenic seedlings of rice under extreme NaCl, PEG, ABA, and dehydration stresses. By combining these approaches, this study intends to explore the following technical and biological questions: (1) is *OsmiR535* involved in the drought and salinity response in rice? (2) What role does *OsmiR535* play in drought and salinity tolerance? (3) Which techniques would be the most effective way to utilize *OsmiR535* to improve drought and salinity tolerance in rice? Our results could provide novel technical and biological insights into the practical application of CRISPR/Cas9-mediated genome editing on drought and salinity tolerance in rice.

## 2. Results

### 2.1. Expression of OsmiR535 in Response to Various Drought and Salinity Treatments

**Figure 1:**
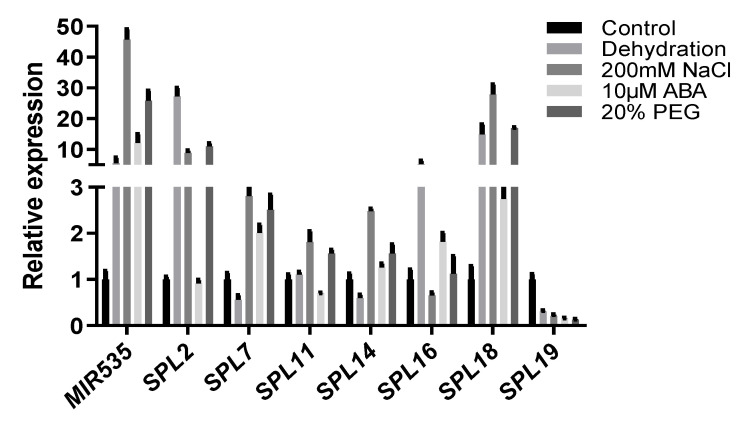
Expression of *OsmiR535* and *OsSPL2/7/11/14/16/18/19* in seedlings under hormone and stress treatments, including dehydration, 200 mM NaCl, 10 μM ABA and 20% PEG. Six seedlings were tested in each treatment, each treatment had 3 replicates, error bars represent the standard deviation (SD). Rice *UBIQUITIN* (*Os03g0234200*) was used as a reference gene.

To validate the involvement of *OsmiR535* in drought and salinity responses, we examined the expression of *OsmiR535* and its predicted target genes *SPL2/7/11/14/16/18/19* under various stress treatments. As shown in Figure 1, the real-time qPCR assay showed that the expression of *OsmiR535* could be induced by dehydration, 200 mM NaCl, 10 μM ABA, and 20% PEG treatments. On the other hand, as the coding sequence of *OsSPL2/7/11/14/16/18/19* have the *OsmiR535* targeting site, their expression level should be inhibited by an elevated *OsmiR535* expression level [10]. However, the expression of *OsSPL2* and *OsSPL18* were upregulated to be 10-fold higher under drought, 200 mM NaCl, and 20% PEG treatments. *OsSPL7/11/14/16* have not displayed a downregulated pattern with elevated *OsmiR535* expression under various drought-related condition. Among the seven target genes of *OsmiR535*, only *OsSPL19* was consistently downregulated under all drought and salinity conditions(Figure 1). Collectively, these results demonstrated that *OsmiR535* is involved in the drought and salinity response in rice, and that *OsSPL19* maybe the main functional *OsmiR535* target gene for the drought and salinity stresses response.

### 2.2. Inhibition and Knockout of OsmiR535 Increased ABA and Salt Tolerance in Rice Seedlings

**Figure 2:**
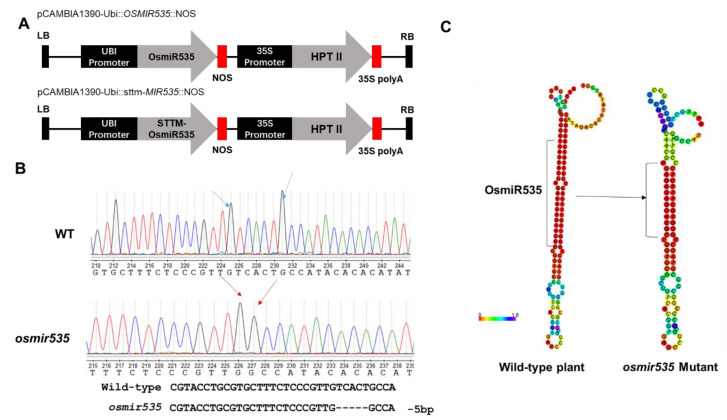
Schematic representation of *OsmiR535*-related constructs and the CRISPR/Cas9 genome editing effect. (**A**) Schematic representation of overexpression constructs *pCAMBIA1390-Ubi::OsMIR535::NOS*, and the *STTM535* construct *pCAMBIA1390-Ubi::* *sttm-MIR535:: NOS.* (**B**) Sequencing diagram of the wild-type (WT) and *osmir535* mutant on the *OsmiR535* genomic region. (**C**) Predicted *OsmiR535* secondary structure of WT and *osmir535*.

To further investigate the role that *OsmiR535* plays in drought and salinity response and tolerance, we generated the CRISPR/Cas9-mediated knockout mutant *osmir535*, overexpression line *OEMIR535,* and *STTM535* transgenic lines (Figure 2A,B). A total of 13 independent transgenic overexpression lines were generated to carry the *pCAMBIA1390-Ubi::OsMIR535::NOS* construct, and T4 and T11 lines were detected as 15- and 20-fold higher than in wild-type (WT) plants, respectively (data not shown). Thus, we chose the T11 line as *OEMIR535* for further investigation. In knockout mutant *osmir535*, a homozygous 5 bp deletion was generated using the pYLCRISPR/Cas9Pubi-N system (Figure 2B) [11]. As shown in Figure 2C, the 5 bp deletion in the *OsmiR535* coding sequence disrupted the second structure of *OsmiR535* and its function (Figure 2B,C) [12].

**Figure 3:**
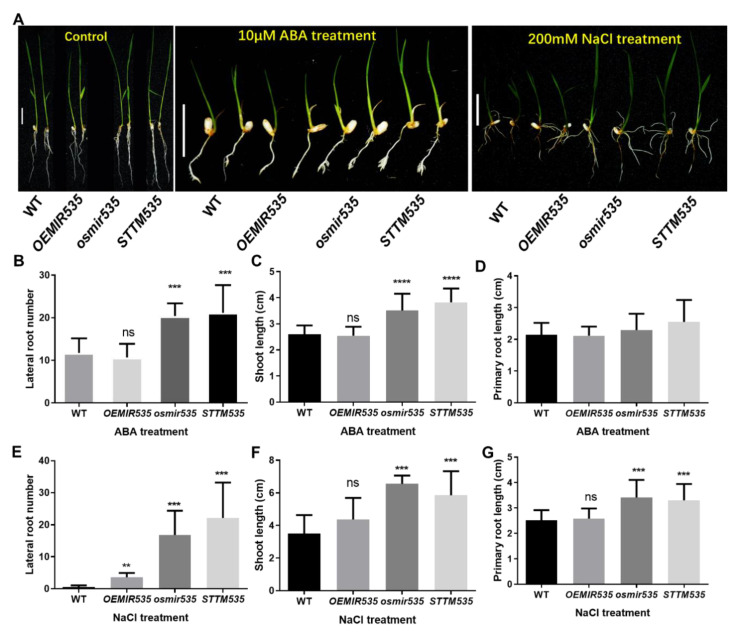
External ABA and NaCl stress testing of *OsmiR535* overexpression, STTM inhibition and CRISPR/Cas9 knockout seedlings. (**A**) 10 μM ABA and 200 mM NaCl treatment of WT and transgenic lines. Lateral root number (**B**), shoot length (**C**), and primary root length (**D**) of WT and transgenic lines under 10 μM ABA treatment. Lateral root number (**E**), shoot length (**F**) and primary root length (**G**) of WT and transgenic lines under 200 mM NaCl treatment. Three replicates of 15 plants were treated. Error bars represent the SD. “ns” represents no significance; One asterisk(*), double asterisk(**), triple asterisk (***) or quadruple asterisk (****) represents *p* < 0.05, *p* < 0.01, *p* < 0.001 or *p* < 0.0001 or statistical difference from the controls, respectively.

To check *OsmiR535* function in ABA- and NaCl-induced stress tolerance at the seedling stage, we applied the external treatment to WT, *osmir535*, *OEMIR535*, and *STTM535*. Among these ABA-treated plants, the lateral root number of the *osmir535* mutant and *STTM535* transgenic plants was 73% and 80% more than WT plants, respectively (Figure 3A,B). Also, the shoot length of the ABA-treated os*mir535* mutant and the *STTM535* transgenic plants was 30% and 47% higher than WT plants. However, both the lateral root number and shoot length of *OsmiR535* overexpression lines were not significantly different compared to WT (Figure 3C). The primary root length of all lines were also not significantly different between the treated different genotypes (Figure 3D), which means that *OsmiR535* functions in modulating the lateral root and shoot growth, but not primary root growth, under the ABA signaling pathway.

On the other hand, the inhibition and knockout of *OsmiR535* also significantly increased the tolerance to 200 mM NaCl treatment by having a positive effect on lateral root number, primary root length, and shoot length in rice (Figure 3E–G). Among the four treated lines, the shoot length of the *osmir535* mutant and *STTM535* transgenic plants was 86.8% and 66.72% higher than WT plants, respectively, while the primary root length was 35.8% and 31.3% higher than WT plants, respectively (Figure 3F,G). On the contrary, the overexpression line *OEMIR535* was not significantly different for both parameters (Figure 3F,G). For lateral root numbers, *OEMIR535*, *STTM535*, and *osmir535* mutants were significantly higher than WT, but the lateral root numbers of *STTM535* and *osmir535* mutants were 366% and 514% higher than *OEMIR535* plants, indicating that the inhibition and knockout of *OsmiR535* enhanced salinity tolerance based on lateral root development. These results demonstrated that either the mutation or inhibition of *OsmiR535* could enhance tolerance of rice seedlings to ABA and salt stresses.

### 2.3. OsmiR535 Overexpression in Plants Causes Sensitivity to PEG-induced Drought Stresses

To test the PEG-induced drought response of WT, *OEMIR535*, *osmir535*, and *STTM535* plants, two-week-old seedlings were transferred into Yoshida solution with a final concentration of 20% (*w/v*) PEG4000 for 10 days to induce PEG stress, and growth for another 10 days in normal Yoshida solution for recovery.

**Figure 4:**
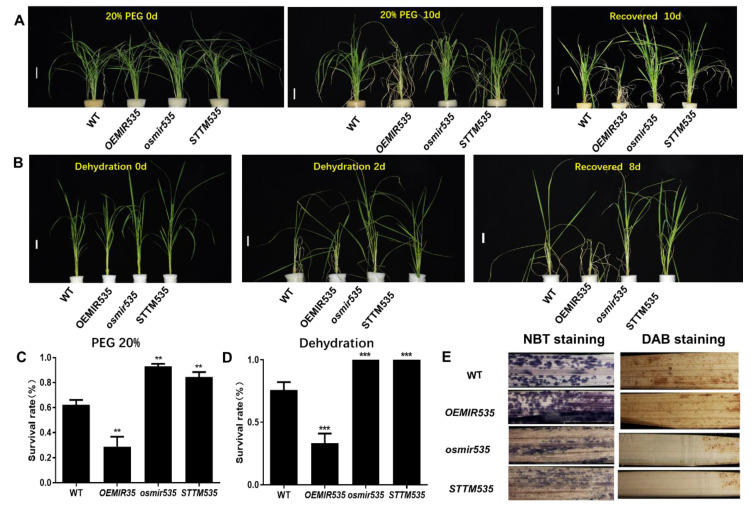
Identification and drought-tolerance tests of *OsmiR535* overexpression, STTM inhibition, and CRISPR/Cas9 knockout plants. (**A**) The phenotype of rice seedlings grown in hydroponic solutions containing 20% PEG for 10 days and recovery for 10 days. (**B**) The phenotype of rice seedlings grown after 2 days of dehydration and recovery for 8 days. (**C**) The survival rate (%) of WT and transgenic lines after 10 days of PEG-induced stress recovery. (**D**) The survival rate (%) of WT and transgenic lines after 2 days of dehydration stress recovery. (**E**) NBT (Nitroblue tetrazolium) and DAB (3,3-diamino benzidine) staining of dehydration-stressed plant leaves. One asterisk (*), double asterisk (**), triple asterisk (***) or quadruple asterisk (****) represents *p* < 0.05, *p* < 0.01, *p* < 0.001 or *p* < 0.0001 or statistical difference from the controls, respectively.

As shown in Figure 4A, the leaves of all four treated lines were rolled and yellowish after 10 days of PEG treatment. *OEMIR535*, the *OsmiR535* overexpression line, displayed the most severe and accelerated senescence phenotype after 10 days of PEG treatment, while the leaves of *osmir535* and *STTM535* plants remained green and alive. Therefore, after 10 days of recovery, it was clear that more leaves of *osmir535* and *STTM535* plants survived under PEG-induced drought stress as compared to WT plants (Figure 4A). On the other hand, after 10 days of recovery, more seedlings of *osmir535* and *STTM535* plants than WT survived after exposure to PEG stresses for 10 days (Figure 4C). Compared to the 62.2% survial of WT plants, 93.0% and 84.3% survial rates were noted for *osmir535* and *STTM535* plants, respectively (Figure 4C). However, only 28.9% of *OEMIR535* plants survived under PEG-induced drought stress (Figure 4C). These results indicated that the overexpression of *OsmiR535* makes plants more sensitive to PEG-induced drought stress, and the inhibition or mutation of *OsmiR535* could make rice seedlings more tolerant to PEG-induced drought stress.

### 2.4. CRISPR/Cas9 Mediated Knockout of OsmiR535 Increased the Survival Rate of Rice Seedlings after Dehydration Stress

To further examine the function of *OsmiR535* in drought tolerance, we performed a dehydration–recovery assay on WT, *OEMIR535*, *osmir535*, and *STTM535* plants. Two-week-old seedlings grow in Yoshida solution were air-dried for two days (dehydration), then transferred into Yoshida solution for eight days (recovery). As shown in Figure 4B, all four treated lines were rolled and yellowish after two days of dehydration. After eight days of recovery, all *osmir535* and *STTM535* seedlings survived, while the survival rate of WT and *OEMIR535* seedlings was 75.8% and 33.3%, respectively (Figure 4D). Nitroblue tetrazolium (NBT) and 3,3-diaminobenzidine (DAB) staining results for all four lines after two days of dehydration demonstrated that unlike both *osmir535* and *STTM535* which produce much less H_2_O_2_ and O_2_ with respect to WT, *OSMIR535* overexpression lines accumulated much more H_2_O_2_ and O_2_, resulting in more susceptibility towards dehydration(Figure 4E). These results are consistent with our results for PEG-induced drought. It is clear that the overexpression of *OsmiR535* makes plants more sensitive to drought stress while its inhibition or mutation makes them more tolerant.

## 3. Discussion

Drought causes multiple negative impacts on plants, such as reactive oxygen species (ROS) accumulation, abscisic acid (ABA) induction, stomatal closure, and retarded growth. Thus, identifying drought-resistance genes could be beneficial for understanding the genetic mechanism of drought tolerance. *OsmiR535* is a single-copy microRNA gene belonging to the *miR156/529/535* superfamily in rice [6,10]. These microRNAs modulate plant development and stress responses mainly through interactions with target genes, leading to either translational inhibition or gene silencing. The *miR156/529/535* genes are well-reported in the complex regulation of SPLs and plant development and architecture [13]. As the sequence shared by *miR156/529/535* is highly conserved, seven SPLs were predicted as common target genes of *OsmiR535*, *OsmiR539*, and *OsmiR156* [13,14]. Although *miR156/miR529/535* were predicted to share a common set of target SPLs, the expression pattern of *miR156/miR529/535* and how they respond to drought stresses are quietly divergent [15]. The expression of *OsmiR156* did not show any significant changes in drought treated tissues [15], and the expression of *OsmiR529* was reported to be downregulated under drought stresses in shoot tissues [16].

In this study, we reported the involvement of *OsmiR535* in drought and salinity response and tolerance. Our qPCR results demonstrated that the expression of *OsmiR535* could be induced by drought and salinity conditions, such as external NaCl, PEG, dehydration, and ABA treatment. This indicated that *OsmiR535* is involed in the drought and salinity response in rice. On the other hand, as there were seven common predicted target genes of *OsmiR156/529/535*, we tested which SPLs may be the key target gene involved in the drought and salinity response. Our results suggest that *OsSPL19* maybe the main functional *OsmiR535* target gene responsible for drought and salinity stress response and tolerance.

To investigate the role of *OsmiR535* in drought and salinity tolerance, we generated a series of transgenic and mutant lines with CRISPR/Cas9, STTM (Short tandem target mimic), and UBI (Ubiquitin) promoter-driven overexpression techniques. We tested the growth parameters of these transgenic and mutant lines under external 200 mM NaCl and 10 μM ABA treatment, and our results demonstrated that either the inhibition or knock-out of *OsmiR535* could significantly enhance the tolerance of rice seedlings to ABA and salinity stresses. Furthermore, we also tested the survival ability of these plants under extreme PEG-induced and dehydration-induced drought stresses. These reulsts showed that both STTM inhibition and CRISPR-Cas9 mediated knockout of *OsmiR535* significantly increased the survival rate of seedlings under extreme PEG and dehydration treatments, while the overexpression of *OsmiR535* made plants more vulnerable to such stresses compared to WT.

## 4. Materials and Methods

### 4.1. Plant Growth Conditions

Rice (*Oryza sativa L.* ssp. *japonica* ‘Nipponbare’) was used for physiological experiments and genetic transformation in this study. Rice seeds were surface-sterilized with 10% sodium hypochlorite solution for 20 min, followed by three rinses with sterile distilled water. Seeds were germinated on wet filter paper for 2 days at 28 °C. Uniform seedlings were selected and transferred to black plastic buckets containing Yoshida solution or agar plates for further treatment. The plants were grown at 28 °C with 16/8 h light/dark cycles, 80% illumination, and 75% relative humidity in a greenhouse. To test plant growth under drought and salinity stresses, we chose 200 mM NaCl, 10 μm ABA, 20% PEG and 2 days air-dry dehydration for the external treatments [17,18,19]. For the 10 μM ABA and 200 mM NaCl treatments, rice seedlings were grown in 1/2 MS (Murashige & Skoog Medium) agar plates for 1 week after germination, then transferred into 1/2 MS agar plates containing 10 μM ABA and 200 mM NaCl, respectively. For PEG tolerance tests, rice seedlings were grown in black plastic buckets containing Yoshida solution for 2 weeks after germination, then transferred into Yoshida solution containing 20% PEG for 10 days, then transferred back into normal Yoshida solution for 10 days. For dehydration tests, rice seedlings were grown in black plastic buckets containing Yoshida solution for 2 weeks after germination, then transferred into an empty bucket for dehydration for 2 days in a greenhouse, then transferred into normal Yoshida solution for 8 days [20]. Survival rates were calculated as survival rate = (survived number of plants after post-stress recovery) / (total treated plants) * 100%. Each treatment was conducted with three parallel replicates.

### 4.2. Vector Construction and Rice Transformation

Full-length pre-miR535 cDNA was cloned into pCAMBIA1390-UBI::NOS (nopaline synthase terminator) to generate the pCAMBIA1390-UBI:*OSMIR535*:NOS overexpression construct (Figure 2A). The short tandem target mimic (STTM) constructs were designed, and primers were synthesized according to a previous study; the amplified STTM region was then cloned into pCAMBIA1390-UBI::NOS to generate the pCAMBIA1390-UBI:*sttm-MIR535*:NOS construct [21]. A CRISPR/Cas9 mediated knockout system was designed and constructed according to the pYLCRISPR/Cas9Pubi-H system manual [11]. All sequenced plasmids were introduced into the rice genome via *Agrobacterium tumefaciens* EHA105-mediated transformation, as previously described [20]. All primers used in this study are listed in Table 1.

### 4.3. RNA Extraction, cDNA Synthesis, and Quantitative Real-Time RT-PCR (qRT-PCR)

Total RNAs were extracted from the fresh roots of WT plants under normal and stress conditions using RNAiso reagent (Takara). Reverse transcription (RT) was performed using PrimeScript™ Reverse Transcriptase (Takara) according to the manufacturer’s instructions. Quantitative real-time RT-PCR analysis was conducted with the Lightcycler 480 machine using AceQ qPCR SYBR Green Master Mix (Vazyme). *UBIQUITIN* (*Os03g0234200*) mRNA was used as an internal control. The specific primers for quantitative real-time RT-PCR are listed in Table 1.

### 4.4. Histochemical Analysis of H_2_O_2_ and O_2_^-^ by 3,3-diaminobenzidine (DAB) and Nitroblue Tetrazolium (NBT) Staining

The accumulation of H_2_O_2_ was determined by staining the leaves of 2-day dehydrated transgenic and WT plants with vacuum infiltration of 3,3-diaminobenzidine (DAB) solution; detailed methods have been described previously [22]. The accumulation of O_2_ was identified by staining the leaves of 2-day dehydrated transgenic and WT plants with vacuum infiltration of nitroblue tetrazolium (NBT) solution, according to the method described previously [23].

## 5. Conclusions

Our results clearly demonstrate that *OsmiR535* responds to external drought and salinity signals, and plays a negative role in drought and salinity tolerance in rice. Morever, as the *osmir535* mutant provides a successful example of generating a drought-tolerant mutant via CRISPR/Cas9 mediated knockout techniques, we propose that *OsmiR535* is a potential genetic editing target for drought and salinity stress tolerance for breeding in *Oryza sativa*.

## Figures and Tables

**Table 1:** The primers used in this study.

Primer	Sequence (5′–3′)	For Experiment
KpnI-MIR535-F	ATAGGTACCGAGGGAGAGAAGAGAGGACACA	Overexpression
BamHI-MIR535-R	CGCGGATCCCAATAAGAGAACATTTAGGGGA	
miR535gRT1	CTCACCGTGACCGCCGCACGTTTTAGAGCTAGAAAT	CRISPR/cas9
OsU6aT1	GTGCGGCGGTCACGGTGAGCGGCAGCCAAGCCAGCA	
miR535gRT2	GCACGCAGGTACGCCGCCGGTTTTAGAGCTAGAAAT	
miR535OsU3T2	CGGCGGCGTACCTGCGTGCTGCCACGGATCATCTGC	
KpnI-STTM535-F	CAGGGTACCTGACAACGAGACTAGAGAGCACGCGTTGTTGTTGTTATGGTCTA	STTM
BamHI-STTM535-R	CGCGGATCCGCGTGCTCTCTAGTCTCGTTGTCAATTCTTCTTCTTTAGACCAT	
qOsUBQ-F	GAAGGAGGAGGAAATCGAAC	Realtime-qPCR
qOsUBQ-R	CTTCACAGAGGTGATGCTAAGG	
qOsSPL2-F	CGTGTTCCAAGAGCCGTACTA	
qOsSPL2-R	GCAGTGGTAGTGGCAGATTTT	
qOsSPL7-F	CGCTCCAGGGAGTCAAGGAG	
qOsSPL7-R	GAGCACGTGGAACCGGCTGC	
qOsSPL11F	GCAGATGCATCTGAAACTGC	
qOsSPL11R	GGTACACTGGGCAATGTGCTGT	
qSPL14F	TCCATTTTGCAGGTCCGGACG	
qSPL14R	GATGCTTGCTGTACAGGATC	
qSPL16-F	TCTCTGCCGTTCTCATGGCAG	
qSPL16-R	CTTACCATGGCAGAAAAGAAC	
qSPL19F	GAAGATGGAGCCAACATTACC	
qSPL19R	GATTCTGCTGAAAGAAGAGGGG	
qSPL18F	CAAGATGTTCTCCTCCGACG	
qSPL18R	AGTTGGCCGGGGATGACA	
qMIR535-F	GGCGGTGACAACGAGAGAGA	
qMIR535-R	GCAGTGACAACGGGAGAAAG	
